# Case of Transfusion

**Published:** 1827-12

**Authors:** R. P. Philpott

**Affiliations:** Brighton


### TRANSFUSION.

*Case of Transfusion; contained in an Extract of a Letter from* Mr. R. P. Philpott, *of Brighton, to* Dr. Blundell. (Communicated by C. Waller, Esq.)

July 21, 1827.—A poor woman, named Ashdown, aged twenty-nine, now in the sixth month of pregnancy, who during three former pregnancies had suffered from varicose veins of the legs, had this day, after standing for some hours at the ironing-board of a laundress, a sudden effusion of blood from the bursting of the vena saphena, about six inches above the right ankle.

About a quarter of an hour elapsed before I reached the house where she was, when she had lost, as it seemed, between eight and ten pints of blood. Assisted by my friend Mr. Phillipson, I, by compress and bandage, immediately stopped the hemorrhage. The patient was in a state of complete syncope, without pulse at the wrist, and but a very indistinct pulsation at the region of the heart; she was cold, with lips and face completely blanched; a profuse sweat broke out, and she passed both fæces and urine involuntarily, so that she appeared moribund. Stimulants applied to the nostrils produced no excitement, and she had totally lost the power of deglutition. Though with little hope or probability of her recovery, we determined to attempt the operation of transfusion.

A person being quickly obtained from whom blood was drawn, a ligature was applied on the right arm of the patient, the median vein laid bare, and carefully dissected, so that a probe was passed under it; the vein was then opened, and the end of a common bone syringe (the only one at hand), with its extremity filed off, was with some difficulty introduced. Several syringes-full were thus injected. When about four ounces had been thrown in, she became extremely restless, throwing her head from side to side, and making an effort to vomit. We then desisted, and had the satisfaction of distinguishing the pulse at the wrist, though extremely small and rapid: warmth was applied, and wine and brandy-and-water administered frequently.

In the evening, six hours after the operation, the pulse was then distinct, and at 120. She was so much better as to answer several questions. Heat was gradually developed, and the next day she was removed in a chaise to her own house.

July 25th.—Slight suppuration became established in the wound in the arm, without the least appearance of inflammation in the vein, although, from the frequent introduction of so rude an instrument, we were afraid the internal coat of the vein might be injured.

30th.—Much better. Has felt no movement of the fœtus since the accident.

August 13th.—She was taken in labour, and in a few hours the fœtus, in a putrid state, expelled. A cool regimen, with light but nourishing diet, and the occasional use of a mild laxative, were the means employed to promote her recovery, and she is now (October 12th) in good health, and following her former avocations.

The ruptured vein in the leg speedily healed, and all trace of the disease has disappeared since her labour. Dr. Blair was kind enough to visit the poor woman several times during her recovery.

*October 13th.*

